# Items of Interest

**Published:** 1895-03

**Authors:** 


### ITEMS OF INTEREST.

Tuberculin is being experimented with as a cure for insanity.

A bill has been introduced in the State Legislature of Pennsylvania to legalize the dehorning of cattle.

The enormous weight of two tons has been reached in a steer of the Durham breed at five years of age.

A bill has been introduced in the New York State Legislature to exempt veterinarians frum jury duty, owing to the character of their professional duties.

One of the leading papers of France strongly urges against the importation of American-beef products. This means the deprivation of their people from the best and cheapest products, and would deny them of the healthiest market of the world.

Some fifty-four students are taking the course of instruction at the Dairy School connected with the Pennsylvania State Agricultural College at Bellefonte.

The Board of Health census of the stables of New York City show the existence of 4380, stabling 69,212 horses, 80 of which contain over 100 each; 920 from 5 to 10 each.

A bill has been introduced in the Legislature of Pennsylvania to prevent the docking of horses' tails, making the same a misdemeanor.

A decree prohibiting the importation until further orders of American cattle into France has just been received by cablegram. This is another blow to American cattle interests.

The Western Humane Society of Pittsburg, Pa., recently had a well-known veterinary surgeon of that city arrested for cruel abuse of a horse which he was called to treat professionally for a defective condition of the teeth. We hope a thorough investigation will prove that there was no ground for such action.

The *Boston Herald* of January 21st contains the most exhaustive practical review of bovine tuberculosis, its relation to public health, measures for its eradication and control, the work done in Massachusetts, with a complete answer of many criticisms made, that has appeared in any public journal in this country.

The agricultural appropriation bill at Washington asks for a much larger appropriation for meat inspection, with a view of extending the work in this direction. It is hoped that in this desire the Bureau of Animal Industry will have the earnest support of every member of Congress, on the broad ground of its sanitary value to our people, as well as the strong tendency it will have to refute the unjust restrictions and untrue criticisms made against our meat products abroad.

At the recent Women's Congress at Washington a paper on "Humanitarianism in Education," by Mrs. Caroline E. White, of the American Anti-vivisection Society, raised a very heated and wide-range argument. The dissection of cats and frogs in public schools for the purpose of teaching physiology, the docking of horses' tails, the wearing of sealskin coats, and heads and feathers of birds, all came in for a thorough discussion, and as a result the house seemed divided against itself.

One authority estimates that the shrinkage in the valuation of the horses of the United States in 1894 reached the gigantic sum of $25,000,000, and from the time that the depressed value of horses set in some three years ago there has been a shrinkage of $64,000,000, which all veterinarians can appreciate by the lessened professional work, especially this year.

Every leading newspaper of Philadelphia has indorsed in its editorial columns the establishment of a Department of Agriculture in Pennsylvania, with a central head in the Secretary of Agriculture, around which a number of departments now in existence may be centrally and effectively associated. Instead of employing, as required, a veterinarian, there will be associated with official power a State Veterinarian, with a liberal salary sufficient to employ a well-equipped man, and thus lead to a more thorough and intellectual direction of the management of the diseases of live stock and their relation to public health.

President Cleveland has just vetoed the act of incorporation bill for American florists. In denying them a national charter he says that State laws are ample for their purpose, and the creation of such a corporation by a special act of Congress would establish a vexatious and troublesome precedent. The bill fixed no limit to the value of real and personal property which the proposed corporation may have held if acquired by donation or bequest. While the promoters of the bill are florists, the bill was drawn to cover horticulture in all its branches.

The bill in Pennsylvania establishing a Department of Agriculture has become a law through the signature of the Governor. Already there have been announced the names of some seven applicants for the position. The law requires a graduate of a legally incorporated and regularly organized college. Drs. Leonard Pearson, M. E. Conard, W. T. S. Werntz, and H. W. Turner, graduates of the Veterinary Department University of Pennsylvania; Drs. W. L. Zuill, Chas. E. Bridge, graduates of the American Veterinary College; Dr. E. E. Tower, graduate of the National College of Veterinary Surgeons, and one from Erie, Pa., name not given, but understood to be a Toronto graduate.

The Inspector-General of the United States Army in his report to General Schofield has this to say: The system under which horses are now supplied has received a fair trial and has been more than once condemned. Nothing, he says, has been done to improve the quality and selection of the horses. He says they should be better bred and conform more to the adopted color. He says that vicious and untrained animals should be no longer purchased, and that it should be no longer the case, as at present, that in every regiment of cavalry there are one or more horses eating their heads off and rendering no adequate return to the Government. During the year 588 cavalry horses have been condemned for various causes. The present low price of horses affords an admirable opportunity to make these much-desired changes.

The most unfair, unjust, and uncalled-for criticisms, garbled statements, inconsistent deductions, have appeared in the "Farm and Dairy" columns of the Philadelphia *Ledger*, to retard and obstruct the efforts of the veterinarians of Pennsylvania to gain a proper, just, and deserved recognition in the counsels of that State in considering the all-important questions as to the best methods of dealing with the contagious and infectious diseases of live stock. No public journal can afford to assume such an attitude, and must always suffer in the public esteem which such journals always hope to win and maintain. The serving of the selfish ends of no one individual can ever justify such actions, much less command the support and influence of a growing profession second to none in importance to the health, wealth, and happiness of the people in every staff and station of life.

As we go to press information reaches us of the final passage of bill No. 24 in the Pennsylvania State Legislature, establishing a Live Stock Sanitary Board, to be composed of the Secretary of Agriculture, the Dairy and Food Commissioner, and the State Veterinarian. This will prove an important step in Pennsylvania, and means much for the better protection of all the interests of the agriculturist, dairyman, and consumer of dairy products.

Later advice reaches us of the appointment by Governor Hastings of Dr. Leonard Pearson as State Veterinarian. Without any disparagement of any of the other candidates for the position, this appointment will meet with the generous approval of every veterinarian in Pennsylvania, and the State thus commands the highest and best talent, and places its seal of recognition upon the proper basis of merit and fitness for the place.

